# The Proceedings of the International Hahnemannian Association; the First Meeting

**Published:** 1881-07

**Authors:** Edward Rushmore

**Affiliations:** Secretary pro tem.


### HOMŒOPATHIC PHYSICIAN.

A MONTHLY JOURNAL OF MEDICAL SCIENCE.

"If our school ever gives up the strict inductive method of Hahnemann, we are lost, and deserve to be mentioned only as a caricature, in the history of medicine."—CONSTANTINE HERING.

Vol. I. JULY, 1881. No. 7.

### THE MEETING OF THE INTERNATIONAL HAHNEMANNIAN ASSOCIATION.

THE first regular session of this Association was held at Brighton Beach Hotel, Coney Island, June 14th–16th.

There were present, of those who assisted in the formation of the Association last year, at Milwaukee, Drs. P. P. Wells, Ad. Lippe, T. F. Pomeroy, Geo. F. Foote, C. Pearson, J. P. Mills and Edward Rushmore. The meeting was called to order by the President, Dr. P. P. Wells; in the absence of the Secretary, Prof. H. C. Allen, Dr. Edward Rushmore was chosen Secretary *pro tem.*

The President delivered an exceedingly able address, on "*The Philosophy of Homœopathy.*" On motion, the thanks of the Association were voted the President for his address, and it was ordered to be printed.

The election of new members being in order, the following gentlemen were elected to membership:

| | |
|---|---|
| DR. E. BAYARD, . . . . . . . | New York. |
| " R. H. BEDELL, . . . . . . | Tremont, N. Y. |
| " J. B. BELL, . . . . . . | Boston, Mass. |
| " J. A. BIEGLER, . . . . . | Rochester, N. Y. |
| " T. P. BIRDSALL, . . . | Wappinger's Falls, N. Y. |
| " T. L. BROWN, . . . . . | Binghamton, N. Y. |
| " F. BRUNS, . . . . . . . | Boston, Mass. |
| " C. W. BUTLER, . . . . . . | Montclair, N. J. |
| " E. CARLETON, JR., . . . . | New York. |

| | |
|---|---|
| Dr. Geo. H. Clark, . . . . . | Philadelphia. |
| " J. B. Gregg Custis, . . . . | Washington, D. C. |
| " J. L. Dunn, . . . . . . | Titusville, Pa. |
| " Adolph Fellger, . . . . . | Philadelphia. |
| " Wm. Jefferson Guernsey, . . | Philadelphia. |
| " R. R. Gregg, . . . . . . | Buffalo, N. Y. |
| " Jno. F. Griffin, . . . . | Williamsport, Pa. |
| " John Hall, . . . . . . | Toronto, Canada. |
| " Horace Hatch, . . . . . | Washington, D. C. |
| " J. R. Haynes, . . . . . . | Indianapolis, Ind. |
| " Walter M. James, . . . . . | Philadelphia. |
| " W. H. Kern, . . . . . . | M'Keesport, Pa. |
| " E. J. Lee, . . . . . . | Philadelphia. |
| " Constantine Lippe, . . . | New York. |
| " J. F. Miller, . . . . . . | Newark, N. J. |
| " Laura Morgan, . . . . | San Francisco, Cal. |
| " E. B. Nash, . . . . . . | Cortland, N. Y. |
| " H. I. Ostrom, . . . . . . | New York. |
| " G. Pompili, . . . . . . | Rome, Italy. |
| " L. A. Rendell, . . . . . | New Haven, Conn. |
| " John C. Roberts, . . . . | New Utrecht, N. Y. |
| " Julius Schmitt, . . . . . | Rochester, N. Y. |
| " Thos. Skinner, . . . . | Liverpool, England. |
| " C. Carleton Smith, . . . . . | Philadelphia. |
| " Samuel Swann, . . . . . | New York. |
| " L. B. Wells, . . . . . . | Utica, N. Y. |
| " Wm. P. Wesselhoeft, . . . . | Boston. |

The following resolutions were offered by Dr. Pearson, and after discussion, adopted:

*Resolved,* That no paper shall be read before this Association, or published in its proceedings, unless the author be a member of the Association, or by unanimous consent.

*Resolved,* That authors be allowed to publish their papers *after* presentation to this Association, if so desirous.

*Resolved,* That in incurable cases, we believe, the utmost possible relief from suffering is obtained from the *Simillimum*, and that antipathic or allopathic palliatives are not only unnecessary, but injurious.

[Presented by Dr. Berridge.]

On motion of Dr. T. F. Smith, the President appointed, as Committee on Publication: Drs. Adolph Fellger, George H. Clarke and E. J. Lee. The committee was empowered to revise the papers, presented before the Association, for publication in THE HOMŒOPATHIC PHYSICIAN.

Dr. Ad. Lippe was elected Chairman of Board of Censors, and authorized to appoint the four members of the board.

Dr. Pearson moved a committee of five be appointed to select device for seal and style of diploma of membership. Motion withdrawn, as Dr. S. Swan generously offered to have designs prepared, and, if approved of by two-thirds of the Association, to have them executed at his expense.

On motion of Dr. Pearson, it was:

"*Resolved*, That we heartily indorse THE HOMŒOPATHIC PHYSICIAN, under the judicious management of Dr. E. J. Lee, as the organ of this Association."

Dr. John F. Miller, of Newark, N. J., gave notice that he would offer a resolution at the next meeting, changing section first of by-laws.

Dr. C. Pearson was elected delegate from the Association to the World's Convention, to be held in London.

The several bureaus then presented their papers; referred to Publishing Committee.

The President appointed the chairmen of the bureaus for 1882 as follows:

BUREAU OF MATERIA MEDICA, ETC.:

Ad. Lippe, M. D., Philadelphia.

BUREAU OF SURGERY:

James B. Bell, M. D., Boston.

BUREAU OF OBSTETRICS, ETC.:

T. L. Brown, M. D., Binghamton, N. Y.

BUREAU OF CLINICAL MEDICINE:

George F. Foote, M. D., Stamford, Conn.

### Officers for 1882.

The following gentlemen were elected officers of the Association for 1882:

President—C. Pearson, M. D., Washington, D. C.
Vice-President—T. F. Pomeroy, M. D., Jersey City, N. J.
Secretary—Walter M. James, M. D., Philadelphia.
Cor. Secretary—E. W. Berridge, M. D., London, Eng.*
Treasurer—Ad. Lippe, M. D., Philadelphia.
Chairman of Board of Censors—Ad. Lippe, M. D., Philadelphia.

On motion of Dr. S. Swan, it was:

"*Resolved*, That the Secretary be instructed to procure from Dr. Allen, the former Secretary, the list of applicants for membership; that the Secretary send a copy of such list, with names of indorsers, to each member of this Association, with the request that they return the same, with their assent or dissent marked opposite each applicant's name; that all those who receive an unanimous vote of assent be considered elected.

The President-elect, Dr. C. Pearson, was authorized to make all necessary arrangements for the next meeting, which will be in Richmond, Va. The following amendments to constitution and by-laws were proposed by Dr. Pearson, for action at next meeting:

#### PROPOSED AMENDMENT TO ARTICLE 3RD OF THE BY-LAWS.

"If an applicant for membership be not elected by the first ballot, he may, upon a majority vote, if he so desires, receive a second ballot at the next annual meeting of this Association."

#### PROPOSED AMENDMENT TO ARTICLE I. OF THE CONSTITUTION.

"This society shall be known as the International Hahnemannian Association, the objects of which are fully set forth in its declaration of principles."

Edward Rushmore, Secretary *pro tem.*

---

The acting-secretary has given us a brief synopsis of the business meetings of the first session of the International Hahnemannian Association. The meeting was in every way a great success. If the Association pursue the course marked out for it in the President's address, there will be a great work accomplished. Let us teach and practice the able and pure homœopathy of Hahnemann, and by so doing we will sooner or later *compel* all, who would have any standing in our school, to join us. Argument will not do this, *but success will.* Editor.

---

* Elected at Milwaukee: continued.

